# Exposure and risk assessment of acetamiprid in honey bee colonies under a real exposure scenario in Eucalyptus sp. landscapes

**DOI:** 10.1016/j.scitotenv.2022.156485

**Published:** 2022-09-20

**Authors:** Nuno Capela, Mang Xu, Sandra Simões, Henrique M.V.S. Azevedo-Pereira, Jeroen Peters, José Paulo Sousa

**Affiliations:** a Centre for Functional Ecology, Department of Life Sciences, Associated Laboratory TERRA, University of Coimbra, Portugal; b Wageningen Food safety Research, Wageningen, the Netherlands; c ForestWISE - Collaborative Laboratory for Integrated Forest & Fire Management, Quinta de Prados, 5001-801 Vila Real, Portugal

**Keywords:** *apis mellifera*, Risk assessment, Field study, Acetamiprid residues, Lateral flow devices

## Abstract

Honey bee colonies have shown abnormal mortality rates over the last decades. Colonies are exposed to biotic and abiotic stressors including landscape changes caused by human pressure. Modern agriculture and even forestry, rely on pesticide inputs and these chemicals have been indicated as one of the major causes for colony losses. Neonicotinoids are a common class of pesticides used worldwide that are specific to kill insect pests, with acetamiprid being the only neonicotinoid allowed to be applied outdoors in the EU. To evaluate honeybees' exposure to acetamiprid under field conditions as well as to test the use of in-situ tools to monitor pesticide residues, two honeybee colonies were installed in five Eucalyptus sp. plantations having different area where Epik® (active substance: acetamiprid) was applied as in a common spraying event to control the eucalyptus weevil pest. Flowers, fresh nectar, honey bees and colony products samples were collected and analyzed for the presence of acetamiprid residues. Our main findings were that (1) acetamiprid residues were found in samples collected outside the spraying area, (2) the amount of residues transported into the colonies increased with the size of the sprayed area, (3) according to the calculated Exposure to Toxicity Ratio (ETR) values, spraying up to 22 % of honeybees foraging area does not harm the colonies, (4) colony products can be used as a valid tool to monitor colony accumulation of acetamiprid and (5) the use of Lateral Flow Devices (LFDs) can be a cheap, fast and easy tool to apply in the field, to evaluate the presence of acetamiprid residues in the landscape and colony products.

## Highlights

• Acetamiprid exposure does not harm honey bee colonies when applied at recommended dose.
• Landscape heterogeneity in forested areas compromises pesticide exposure assessment.
• The increase of the sprayed area leads to higher acetamiprid residues in the colonies.
• Honey bee colony products can be used as a bioindicator of environmental pollution.

## Graphical abstract

**Figure:**
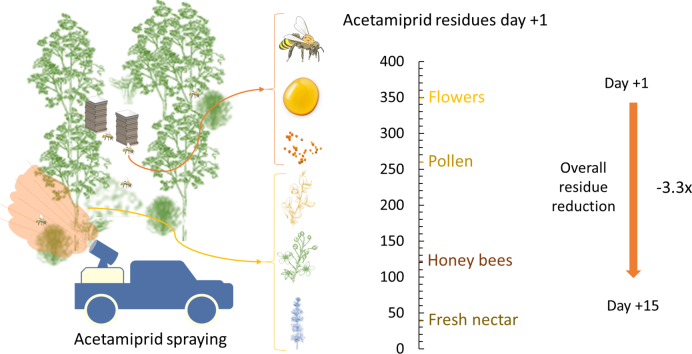

## 1 Introduction

Honey bees are exposed to a wide range of biological, environmental, and chemical stressors (Steinhauer et al., 2018), that can be influenced by colony traits and the landscape where these colonies are installed (e.g., Meikle et al., 2020; Steinhauer and Saegerman, 2021). The landscape surrounding honeybee colonies can be considered as a complex environment, with temporal and spatial shifts generated mostly by human management (in non-natural areas; Tscharntke et al., 2005; Odoux et al., 2014; Requier et al., 2015; Meikle et al., 2020). Intensive agriculture and forestry have been transforming landscapes, comprising intensively managed areas in which wild flowering resources are scarce or might not even exist (Nicholls and Altieri, 2013; Requier et al., 2015). Landscape fragmentation and food scarcity or non-diverse food sources can lead to a poor diet for honeybees. Honeybees need to acquire essential amino acids and proteins for normal larvae development, which can be obtained from different plant species (Brodschneider and Crailsheim, 2010). Low diversity and/or abundance of plant species – thus potentially low diversity of nutrients – might compromise their immunocompetence against pathogens (Alaux et al., 2010). Moreover, modern agriculture (and modern forestry, to a less extent) is highly dependent on the use of plant protection products (PPPs) to prevent production losses due to pests/pathogens. PPP formulations have different modes of action, depending on their active substance, and can be harmful to non-target species like honeybees (Pisa et al., 2015; Hopwood et al., 2016). In fact, these chemicals can act synergistically (e.g., Sgolastra et al., 2017) and negatively contribute to the challenges caused by nutritional stress (Tosi et al., 2017) or by other stressors like the ectoparasitic mite *Varroa destructor* (Straub et al., 2019). To address this problem, pesticide risk mitigation measures aim to limit the exposure of the applied products to non-target organisms (e.g., Regulation (EC) No 1107/2009, 2009). Nevertheless, due to their broad foraging range behavior, bees are commonly exposed to a variety of PPPs (Sanchez-Bayo and Goka, 2014; Simon-Delso et al., 2017; Tosi et al., 2018; Zioga et al., 2020), and several studies have acknowledged a possible link between classes of pesticide exposure (e.g., neonicotinoids) and the decline of bee health (Hopwood et al., 2016; Wood and Goulson, 2017).

Neonicotinoids (e.g., acetamiprid, thiamethoxam, imidacloprid, thiacloprid and clothianidin) have become the most used insecticides worldwide, with a market share of 25 % (Bass et al., 2015). These systemic insecticides remain in the vascular system of the plants for several days, protecting the crop from a variety of pests. Such protection occurs by blocking impulse signals on insects' central nervous system, thus limiting their activity (i.e., paralysis), and therefore causing death (Tomizawa and Casida, 2005). Due to their systemic nature, nectar and pollen of plants treated with these PPPs might also contain the active substance, which can be ingested by insects while feeding (including non-target organisms like honeybees and other pollinators; Simon-Delso et al., 2015; Hopwood et al., 2016). Even so, and despite the long history of usage of neonicotinoid-containing products, most of the studies linking effects of this class of pesticides on colony health were published only after 2011 (Lu et al., 2020). These pesticides show low lethal effects when sprayed and honey bee colony resilience can mask the loss of forager bees (Schmickl and Karsai, 2017). By inducing mainly sub-lethal effects at different stages of honey bee development, the effects at colony level are not immediately detected, being visible only after prolonged exposure (Lu et al., 2012; Rondeau et al., 2014). Over the past years, the use of several pesticides containing neonicotinoids have been severely restricted in the European Union (imidacloprid [EU, 2018/783], clothianidin [EU, 2018/784], thiamethoxam [EU, 2018/785], thiacloprid [EU, 2020/23]), leading eventually to the withdrawal of these pesticides. Besides the emergency authorizations for some neonicotinoids, currently, only acetamiprid is authorized for outdoor crop protection, having been renewed until 2033 (EU, 2018/113). In fact, acetamiprid is considered safer for honey bees as it has a LD_50_ (by contact) 100 times higher than the one reported for thiamethoxam (Lewis et al., 2016) and when compared to this later substance and imidacloprid, is the least transferred neonicotinoid into the hives via pollen and nectar (Niell et al., 2017).

The European Food Safety Authority (EFSA) stated that acetamiprid has “a low risk to bees”, considering that a ban or further restrictions of this substance was not scientifically or legally appropriate (EFSA, 2016). Nonetheless, this decision was based on data from tests developed under laboratory conditions (tier I), since semi-field (tier II) and field (tier III) studies, had major drawbacks: short duration, lack of exposure assessment (unavailability of data on relevant metabolites in pollen and nectar) and low number of colonies used (EFSA, 2016). This implies that there is still a lack of relevant semi-field and field studies regarding exposure pathways and effects of environmentally realistic concentrations of acetamiprid-based insecticides (Heimbach et al., 2017). To tackle the need for field studies (tier III), risk assessment for honeybees at a landscape level rely on exposure predictions based on landscape composition (field sizes and crop attractiveness), sprayed area and honeybee foraging ranges (Appendix E in EFSA, 2013). However, such schemes can be difficult to apply in some landscapes, yet field studies are necessary to infer the real exposure pathways and calculate risks/toxicity.

In Portugal, acetamiprid has been used to control the spread of *Gonipterus platensis* in eucalyptus areas, by spraying the formulated products directly to the tree canopies (Valente et al., 2004). *G. platensis* is a known eucalyptus weevil whose larvae feed on eucalyptus leaves and young shoots. Since eucalyptus trees do not flower during May, they do not provide resources for pollinators, thus one cannot use the landscape level approach used in risk assessment. Possible exposure can derive from flowering plants from the understory vegetation, that can come in contact with the insecticide via aerial drift or wash-off, which makes pollinator exposure hard to predict.

In the present study, we used several study windows dominated by eucalyptus plantations, to capture a range of acetamiprid exposure situations to which colonies might be subjected to. Exposure can be conditioned by the colonies' location ((un)protected by vegetation), wind speed and direction, density of understory vegetation (mainly shrubs) and trees, and also by colonies' foraging area in relation to the sprayed area. We hypothesized that (1) there is a gradient of acetamiprid accumulation in the understory vegetation from the most exposed vegetation (near the roads used for spraying) to the most protected vegetation (inside eucalyptus stands) and (2) apiaries installed in landscapes with larger sprayed area transport more acetamiprid residues into the colony. Our aim was to understand the range of honey bees' exposure to acetamiprid residues under realistic field conditions in this type of forest habitat, and the implications when deriving risk values.

## 2 Materials and methods

### 2.1 Test areas and setting the experimental hives

Five eucalyptus-dominated stands presenting high incidence and prevalence of *G. platensis* were selected in articulation with Altri Florestal®, among the several forest areas managed by this company. In these parcels, the commercial formulation Epik® SG (SIPCAM; 20 % p/p acetamiprid) was applied (during May 2020) to control the eucalyptus weevil (200 g/ha; 40 g a.s./ha). Pesticide application was done by aerial spraying from the existing forest roads, using an ultra-low volume canon (debit of 50 L/h) attached to a pick-up truck, aiming at the top of the trees (where *G. platensis* larvae feed) and always considering the topography of the selected parcel and current weather conditions (e.g., absence of rain in the 24 h after spraying, temperature < 30 °C, and wind speed <11 km/h). From the spraying events done in the selected parcels, five locations (Supplementary material A) were chosen based on the size of the sprayed area (ranging from 0.25 to 4.39 km^2^) to seek a gradient of area of exposure upon which the colonies were foraging on. Each study window comprised an area of 2500 m radius from the central point where the colonies were installed (total foraging area of 19.63 km^2^) and encompassed the parcel that was sprayed; a common feature of all the five study windows is that the colonies were always installed within the sprayed parcel. The total area of the study window was calculated based on foraging information from previous field work in a similar forest habitat (Domingues et al., unpublished). Four of these study apiaries (A1 to A4) were installed within an area of 40 km^2^ while the fifth apiary (A5) was 25 km Northwest.

All the selected study windows (Supplementary material A) are managed forested areas used for intense *Eucalyptus* sp. plantation, intended to provide raw material for paper/pulp industry. Spraying areas were previously defined to guarantee total spraying coverage in the marked area. Non-eucalyptus riparian vegetation neighboring small intermittent streams (dry during the course of the experiment) as well as large forest clearings, were not marked for spraying.

To evaluate the most common flowering resources and their hotspots, a survey of existing flowering species was performed the day before the experiment, by scouting through all the roads inside the spraying area (a band of 30 m to each side was inspected). To assess exposure to acetamiprid due to foraging, potential honeybee resources were collected by random sampling the identified hotspots, considering flowering patches that presented plant species with known honeybee attractiveness (species described in Caravela et al., 2019).

Two days before being transported to the field, colonies were selected among two levels of biological status (weak vs strong colonies), focusing on the major colony traits: population was assessed by weighing all the frames with and without bees, while brood and resources were calculated by photographing the frames and using the DeepBee® software (Alves et al., 2020). The health status was also evaluated, using a list of all the known disease symptoms (Appendix D – EFSA AHAW Panel, 2016), with a special focus given to *Varroa* mites to ensure low levels of infestation (sticky bottom method; Flores et al., 2015). Since colony population size determines colony behavior and interaction with the landscape (e.g., Beekman et al., 2004), one strong (approx. 30,000 bees) and one weak (approx. 20,000 bees) colony were installed inside each of the selected study windows, one week before the spraying event, to allow the colonies to acclimate to the new landscape. After being installed, each colony was equipped with a pollen trap, which was only activated during specific periods for pollen collection. Data about the health/strength of the colonies, before (approximately 1 week) and after (approximately three weeks) the spraying event, can be found on supplementary material (Supplementary material B).

### 2.2 Sampling events

Four sampling events (Table 1) were determined: one day before spraying (day −1) on the day of pesticide application immediately after the spraying event (day 0), one day after pesticide application (day +1) and 15 days after the application (day +15).

**Table 1:** Number of collected samples in all the apiaries/landscapes, during the experiment, and the respective acetamiprid residues measured (mean; min. – max.).

	Type of sample	Before spray Day −1	Spraying day Day 0	After spray Day 1	After spray Day 15
Colonies	Beebread	10 (0)			10 (408; 15–1980)
	Pollen	10 (0)		10 (262; 15–592)	10 (21; 1–70)
	Honey	10 (0)			10 (23; 0–114)
	Bees	10 (0)	10 (359; 41–1000)	10 (123; 4–608)	10 (7; 2–21)
	Fresh nectar			10 (41; 10–180)	
Landscape	Flowers	20 (0)		50 (350; 10–1000)	50 (81; 0–586)
	Leaves	20 (0)			
	Soil	4 (0)			
	Eucalyptus leaves	4 (0)			

One day before the spraying experiment (day −1), beebread, honey, pollen (≥ 5 g) and forager bees (≥ 20 bees) were collected from all colonies. From the surroundings of each apiary, one pooled soil sample (from 4 locations), one pooled eucalyptus leaves sample (from 3 trees), 4 flowers and 4 leaves samples were randomly collected from flowering plants with high beekeeping potential (See Table 1 for the total number of samples). All the samples were collected into glass vials and stored in a cold and dark container (with ice bags), until being frozen at -20 °C in the same day. Sampling site characteristics (distance to closest spraying point [meters], position considering spraying map [mark/unmarked for spraying]) and coordinates were annotated. Before spraying (early in the morning), the colonies were closed (covering the entrance with foam) after some foragers had already left the colony. Immediately after spraying (day 0), all foraging honeybees arriving at – or in the vicinities of – the closed colonies were captured and stored, to account for exposure by contact.

On day +1, a pooled sample of at least 20 forager bees were collected to measure oral exposure using a sweep net. Additionally, one pooled sample of fresh nectar (≥ 2 ml - by gently squeezing forager bees to collect nectar from their honey stomach - Gary and Lorenzen, 1976) and a sample of fresh pollen were collected from each colony, using pollen traps (≥5 g; traps activated from 7/8 a.m. to 6/7 p.m.). In the sprayed area, regions with high potential for honeybee visits (plants with high beekeeping interest) were screened and 10 flower samples were randomly collected from different shrubs. The aim for collecting different types of samples was to gather knowledge on pesticide distribution in the flower resources and its transferability (via pollen and nectar) into the colonies.

On day +15, pesticide residues in flowers from each study window were analyzed by randomly collecting 10 samples of flowers following the same procedure as stated above (day +1). Therefore, flower samples were not necessarily collected from the same plants as before. Pesticide residues in the colony were assessed by collecting a pooled sample of beebread, honey (≥ 5 g of each) and forager bees (≥ 20 bees) that where stored and later analyzed. The flower resources-colony residues transferability was accessed by the analysis of fresh pollen (≥ 5 g) collected from each colony using the pollen traps.

### 2.3 Sample analysis

Samples collected on day −1 (before the spraying event) were analyzed by a qualitative (presence/absence) screening, using a paper-based LFD immunoassay containing specific antibodies for acetamiprid (Wang et al., 2017; Liu et al., 2019). All the samples collected after spraying, from colony and landscape matrices, were analyzed by a matrix-matched semi-quantitative xMAP acetamiprid immunoassay (e.g., Guo et al., 2013; Hamza et al., 2014; Peters et al., 2014). Additionally, a subset of the extracts was also screened using the aforementioned LFDs. More details of the used methodologies can be found in the “Supplementary material C”.

### 2.4 Exposure and risk assessment

Colony's age polyethism can lead to different exposure levels, since honeybees adapt their feeding needs according to the task they perform in the colony (Johnson, 2008; Brodschneider and Crailsheim, 2010; Rodney and Kramer, 2020). Therefore, for each bee class, daily Residue Intake (RI; mg/day) was calculated using the following formula, adapted from EFSA (2013):

$$\mathrm{RI}=\left(R_{\mathrm{pollen}}\times C_{\mathrm{pollen}}\right)+\left(R_{\mathrm{nectar}}\times C\;\mathrm{nectar}\times d_{s}/m_{s}\right)/1000$$

where the R_pollen_ and R_nectar_ are the ‘residues concentrations’ (mg/kg) in pollen and nectar, respectively. C_pollen_ (consumption rates of pollen), d_s_ (mg/day; intake rate of sugar), and m_s_ (kg/kg; sugar content in nectar) were based on the estimations from table J1 in EFSA (2013). Since the sugar concentration in nectar was not measured, a worst-case scenario was assumed and a low threshold value for nectar and honey (20 and 50 %, respectively) was used. Additionally, to calculate larvae exposure, the total consumption of pollen and nectar during the 5 days of larvae development was considered.

Exposure Toxicity Ratio (ETR) values (risk values) were calculated for each colony and for each class of bees (i.e., forager, nurse, winter, and larvae), using the exposure values measured in the present study, and toxicity values obtained from European Commission (2004), and compared with the trigger values (obtained from table 3 in EFSA, 2013). ETR values below the trigger values indicate an acceptable risk.

### 2.5 Statistical analysis

Acetamiprid residues in flowers were compared at several levels via Linear Models (LM) and using log transformed data. LMs were chosen after checking the best distribution fit for each dataset and after analysis, data normality and model fit were checked via Q-Q plots and residue analysis. Comparisons were done in acetamiprid values between the five study windows at day +1, between day +1 and day +15 (considering all study windows), and to assess the effects of distance from the spraying location (nearest road) and if the sample was collected inside an area marked for spraying (spray Y or N) (also considering all study windows).

The relation between the sprayed area and the acetamiprid levels in pollen and nectar at day +1 were analyzed by performing a simple linear regression (nectar residue data was log transformed). Also, across all study windows, differences in acetamiprid values between pollen and fresh nectar at day +1 were compared by a LM using log transformed data. The same method was used to compare pollen residues between day +1 and day +15, and the residues between beebread and honey on day +15.

Analyses were performed with *R Studio version 1.4.1106* using the nlme (Pinheiro et al., 2019) and ggplot2 (Wickham, 2016) packages.

## 3 Results

### 3.1 Flowering resources and acetamiprid residues

In all study windows, the most abundant flower resources with known attractiveness for honeybees (by providing pollen and/or nectar) were *Cytisus striatus*, *Cistus ladanifer*, *Lavandula* sp., *Rubus* sp. and *Echium* sp. *C. striatus* and *C. ladanifer* were only sampled on day +1, as they were already reaching the end of the flowering period (few flowering buds), translating to a lower sampling effort. *Lavandula* sp. and *Echium* sp. were the most abundant flowering resources throughout the experiment, while *Rubus* sp. was flowering only on day +15. None of the samples collected before spraying had any residues of acetamiprid (Table 1).

**Fig. 1:**
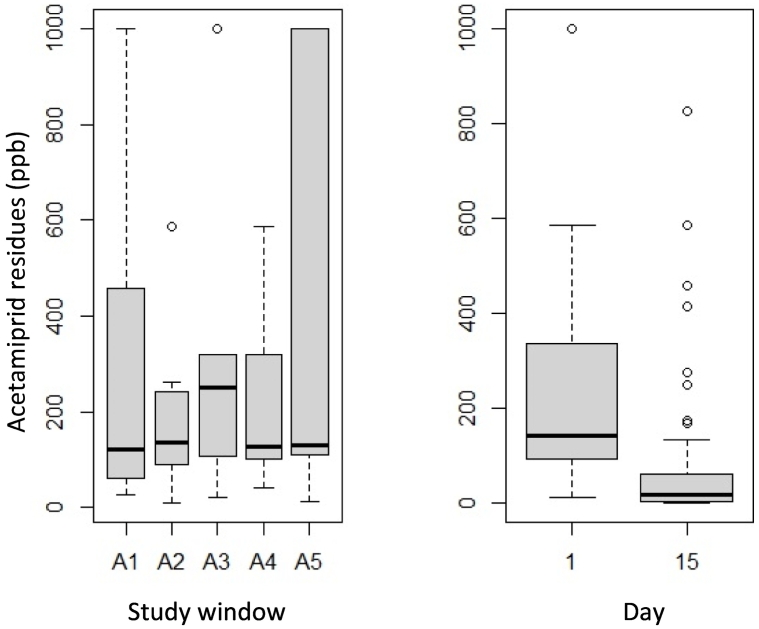
Acetamiprid residues found on flowers on the day +1 within the different apiaries/landscapes (left) and acetamiprid residues on all flower samples from day +1 and day +15 (right).

On day +1 (after spraying), no statistical differences on acetamiprid concentrations in flower samples (*p* = 0.82) were found between the different study windows. Acetamiprid residue concentrations ranged from 11 to 1000 ppb (Fig. 1). Despite the significant reduction in acetamiprid residues from day +1 to day +15 in all areas (p < < 0.001; Fig. 1), almost all flower samples still contained acetamiprid residues in concentrations >1 ppb (Mean = 81 ± 360).

**Fig. 2:**
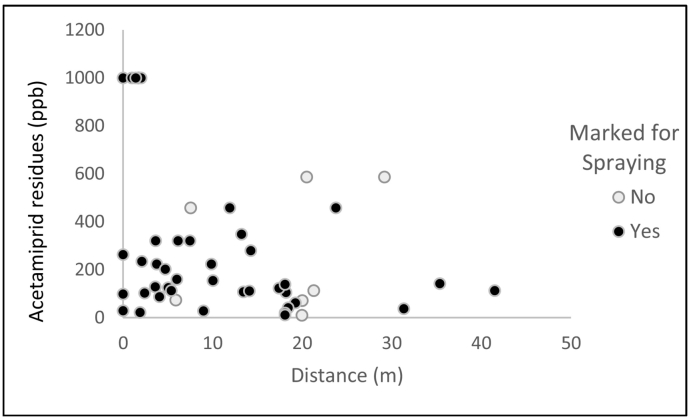
Acetamiprid residues on flowers considering their position regarding the distance from the spraying point (x axis) and if it was previously marked for spraying (black dots).

None of the sampling point characteristics ([1] distance to closest spraying point, and [2] position considering spraying map [mark/unmarked for spraying]) could explain the amount of acetamiprid residues present in the flower samples (*p* = 0.07, and *p* = 0.22, respectively). Even in areas where spraying was avoided (variable: marked/unmarked), acetamiprid residues above 10 ppb were found in all samples in day +1 (Fig. 2).

### 3.2 Acetamiprid residues in the colonies

**Fig. 3:**
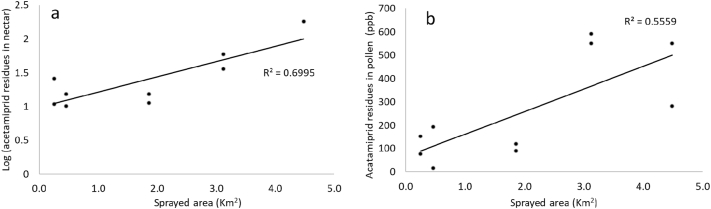
Acetamiprid residues in nectar (a) and pollen (b) samples collected on the day +1, considering the amount of area in which the insecticide was sprayed.

As expected, the chances of foraging on sprayed plants increased with the size of the spraying area (Fig. 3): nectar residues (M = 40.6 SD = 55 ppb) had a strong linear relationship with the area (R^2^ = 0.7, *p* ≤ 0.05, Fig. 3a), while the pollen residues (M = 262.4 SD = 218 ppb) had a moderate positive linear relationship with the size of sprayed area (R^2^ = 0.55, p ≤ 0.05, Fig. 3b). At day +1. the amount of acetamiprid residues on pollen is significantly higher than on nectar (p < <0.001; Table 1). After 15 days, acetamiprid residues in pollen pellets were 12.5 times lower (day +1 M = 262 SD = 218; day +15 M = 21 SD = 18 ppb; Table 1), when compared with day +1 (p < < 0.001).

Despite the reduction of acetamiprid residues in flowers between day +1 and day +15, bees kept collecting resources during this period, and the residues are likely to have accumulated on bee matrices. On Day +15, mean residue levels found in honey (M = 23 SD = 33, from 0.3 to 114 ppb; Table 1) were 17 times lower than the mean residues found on beebread (M = 407 SD = 666, from 15 to 1980 ppb; Table 1) (*p* < 0.01).

### 3.3 Exposure and risk assessment

The tested landscapes allowed us to capture several exposure scenarios (high to low density of eucalyptus foliage protecting the colonies) for forager honeybees and to calculate the amount of active substance that bees might be exposed by contact (forager bees residues after spraying range from 40 to 1000 ppb, mean = 359 ± 368 ppb). Oral exposure of forager honeybees was calculated on day +1 and day +15: forager bees were carrying 17.5 times less residues from day +1 to day +15 (from M = 123 ± 192 to M = 7 ± 5 ppb).

The calculated ETR values were below the trigger values for all the colonies, both considering residue levels found on pollen and fresh nectar on day +1, as well as on honey and beebread on the day +15 (Table 2).

**Table 2:** Exposure Toxicity Ratio (ETR) values for different classes of bees considering their food consumption (nectar and pollen) and the LD/LDD 50 and NOEL for the acetamiprid. Only the highest (from all the colonies) calculated ETR values are reported. If the ETR values are smaller than the trigger value then the specific protection goals are achieved (acceptable risk).

Type of assessment	Endpoint	Class of bee	ETR	Trigger values
Acute oral exposure adult bees	LD50 = 8.85 μg a.s./bee	Forager bees	0.0033	0.2
		Winter bees	0.0016	0.2
		Nurse bees	0.0028	0.2
Chronic oral exposure adult bees	LDD50 = 11.7 μg a.s./bee	Forager bees	0.0025	0.03
		Winter bees	0.0012	0.03
		Nurse bees	0.0022	0.03
Chronic oral exposure larvae	NOEL = 5 μg a.s./bee	Larvae	0.0204	0.2

## 4 Discussion

Landscape heterogeneity (e.g., spatial distribution of the flower resources in the sprayed area), together with the spraying method (e.g., non-uniform pesticide application and drift), could be behind the observed variability in acetamiprid in flower samples, originating no statistical differences between the tested study windows. Fifteen days after the application, the acetamiprid concentration in all the different matrices collected were reduced, in average, by 3.3-fold. Of note, when flower samples were collected for pesticide analysis, preference was given to flowering resources being visited by honeybees at that point in time, thus sampled plant species were not the same between the two sampling periods. Consequently, extrapolations about pesticide degradation in the flower samples should be done with caution.

Management practices in the study windows included the removal of vegetation between eucalyptus lines by plowing. These practices, along with the high tree density, can lead to low flowering resources inside the eucalyptus stands (shrubs were only present in *Eucalyptus* plantation lines). Therefore, most of the flowering resources/sampling points were within the forest clearings and small water channels (Domingues et al., unpublished), and not inside the eucalyptus areas. Despite spraying maps (Appendix B) indicating these sensitive areas were to be avoided (not sprayed), acetamiprid residues were detected on flower samples from these areas (18 % of all sampling points). The unintentional contamination of non-target areas is a common event almost at every pesticide application (Fishel and Ferrell, 2010). To control the level of drift, besides the use of buffer areas there are several other mitigation strategies (e.g., avoid spray with strong wind, use of crop specific delivering methods, use of windbreaks) that can be taken into consideration (Ucar and Hall, 2001). In this study, the spraying event only took place when wind speed was lower than 11 km/h, while the pesticide solution was applied at the top of the eucalyptus trees using a nebulizer cannon that created small droplets with a charge that easily adhere to the eucalyptus foliage, reducing the washoff towards the soil and understory vegetation. This means that even with tight safety spraying measures, implemented to reduce cross-contamination of non-target areas, air drift did occur, resulting in exposure of honeybees due to foraging.

The amount of acetamiprid residues found in the different matrices collected in the sprayed area gives a proxy of how organisms can be exposed. Honeybees forage in these landscapes and bring nectar and pollen (among other elements, like resins and water) into the colonies, either from the sprayed area or outside of it. As the spraying area increased, higher honey bee's exposure was expected and confirmed by an increasing amount of acetamiprid residues found in pollen and nectar with an increase of the area sprayed. This confirms that the acetamiprid concentration found on pollen pellets provides a representative image of flower contamination at a moment in time, making the honey bee colonies good indicators of environmental pollution by xenobiotics (Bargańska et al., 2016; Niell et al., 2018).

Concentrations of acetamiprid found in pollen pellets were much higher than the ones found in nectar one day after spraying (*p* < 0.05). This can be explained by the fact that bees metabolize at least 50 % of acetamiprid in the digestive tract just 30 min after nectar ingestion (Brunet et al., 2005). However, when this nectar is transformed into honey, the acetamiprid degradation is significantly impeded (half-life of 200 days in multiflower honey; Yeter and Aydın, 2020). As for pollen (and beebread), the presence of higher acetamiprid concentrations is possibly related with these matrices' ability to retain both lipophilic and hydrophilic chemicals (Lozano et al., 2019). The samples collected after 15 days agree with these assumptions, considering the higher acetamiprid residue levels found in beebread when compared to honey. In these samples, colonies from the apiary 4 have significantly higher amounts of acetamiprid residues when compared to the other colonies. The landscape surrounding this apiary has several intermittent streams covered with shrubs of *Rubus ulmifolius*, whose flowering period only started after the spraying event. Considering the systemic characteristic of this class of pesticides, it is expected that plants might have been exposed to acetamiprid during the spraying event, with the insecticide entering the vascular system through the leaves/roots, traveling into the nectar and pollen, and thereafter being transferred into the colonies after blooming. This specific case also demonstrates the difficulties in calculating bee's exposure to neonicotinoids in such heterogenous landscapes when considering flower resources (even though all landscapes were mainly composed by eucalyptus plantations).

Taking into account that beebread and honey reserves are commonly consumed by the colonies during the most critical period for survival (i.e. winter; e.g., Brodschneider et al., 2018), that the winter bees live longer than summer bees (as their activity is highly reduced and body fat is highly increased; Amdam and Omholt, 2002), and that at the same time, the colony nest is highly reduced/nonexistent leading to a few/no bees being born, stressors that affect the colony equilibrium are able to cause the colony to collapse (e.g. Van Dooremalen et al., 2012). In previous reports (EFSA, 2013), winter bees, larvae, nurse bees, and foraging bees are the classes of honey bees that are more exposed to pesticides through oral exposure. In this study, for all of these classes, despite assuming the worst-case scenario (e.g., honey with 50 % sugar concentration), the ETR values were considerably lower than the trigger values, even with those having been conservatively calculated based on the lowest background mortality levels found in the literature (EFSA, 2013). This indicates that under these specific conditions, there are no honey bee classes seriously harmed by these spraying events. Other studies have also shown that field applications of acetamiprid did not harm honey bees (no increase in mortality nor behavioral changes) when using the recommended dose (Stanley et al., 2015; Dworzańska et al., 2020). The absence of significant effects on adult bee mortality also occurred in laboratory studies performed by Stanley et al. (2015) when the recommended dose was used. Furthermore, similar doses to the ones found in the fresh nectar do not affect honey bees homing ability (Capela et al., 2022). Negative effects of acetamiprid were only detected on honey bee lifespan and foraging behavior after exposure to 2 μg/a.s./bee (exposure by contact; Shi et al., 2020a), and on larval development after exposure to 5 ppm (oral exposure; Shi et al., 2020b). In the current study, the maximum value detected in colony samples, was 1980 ppb in beebread samples, which is approximately 2.5 times lower than the harmful values reported by Shi et al. (2020b). Interestingly, acetamiprid structure is being used to search for alternative pesticides that are less harmful for honey bees (Crisan et al., 2020).

The wide range of acetamiprid residues found in all sites also shows how extremely difficult it is to predict pesticide exposure in this type of landscapes. The amount of pesticide residues that bees transport into the colony (as nectar and pollen) can be seen as a measure of the real exposure conditions that honeybees might be subjected to. This assessment gains an even higher relevance since food sources vary spatially and seasonally (Berenbaum, 2016), and exposure may depend on the foraging behavior (visited plants). Also, when assessing exposure to honeybees, it can be misleading to only evaluate residue levels immediately after pesticide application. Not only can they be found in plants several days after application, but the collected nectar (as honey) and pollen (as beebread) will also be stored in the colony, thus originating an accumulation of pesticide residues in these matrices for a long time (e.g., Tong et al., 2018), and being an exposure source for bees inside the colony. Therefore, sampling bee products becomes essential to determine the real colony exposure and the effects of the accumulated residues which will later be used to feed future generations (Colin et al., 2019). In this study, the maximum residue levels detected in pollen and nectar were 592 ppb and 180 ppb, respectively, which are much higher than the detected maximum residues in other studies. In a review from Zioga et al. (2020), the maximum residues found in pollen were 0.82 ppb while in nectar where 7.60 ppb. These differences may arrive from the lower application rates (20 g a.s./ha vs. 40 g a.s./ha in our study), timing and sampling methods used in the different studies (not specified in the review) since we measured the fresh nectar and pollen transported by the bees in the day after spraying. When compared to other neonicotinoids, acetamiprid seems to be the least transferred into the colonies. In Niell et al. (2017), acetamiprid residues found in the colony (honeybees) were 10 times lower than the other tested neonicotinoids (thiamethoxam and imidacloprid). The authors hypothesized that bees could detect the presence of acetamiprid due to the high volatility of the insecticide. Nonetheless, acetamiprid repellent effects were detected for termites (Rust and Saran, 2008) while for honey bees there are no evidence of such effects. The available data also confirms that the maximum acetamiprid residues found in colony products (nectar and pollen) have a lower concentration than other neonicotinoids (pollen / nectar maximum residues concentration (ppb) for each neonicotinoid in Zioga et al. (2020): acetamiprid – 0.82 / 7.60; imidacloprid – 159 / 6588; thiamethoxam – 95.2 / 11; thiacloprid – 78 / 65.6; clothianidin – 11 / 2992). Therefore, acetamiprid seems safer than other neonicotinoids due to its lower toxicity and lower transference into the colonies.

Despite the lower toxicity from acetamiprid we advocate that to avoid unacceptable risks derived from pesticide exposure to honey bees (in general), not only should it be considered that mitigation measures may and can be applied to decrease the exposure of non-target plants, instead of just following the “non-attractive crops” requirement (Simon-Delso et al., 2017), but also that collecting residue data from flower resources and from the colony is fundamental to better predict exposure. In this study, eucalyptus trees (the sprayed “crop”) were not flowering, which might lead one to infer that spraying events in these areas and during this season can be safe for pollinators when considering oral exposure. Nonetheless, under the sprayed “crop” and next to it (surrounding areas), there are important food sources and even nesting places that sustain pollinator communities. The acetamiprid residues found on the analyzed matrices show that these sources also get pesticide residues input and that honeybees transport those xenobiotics into the colonies. This makes the honeybee colonies good indicators of environmental pollution by xenobiotics (Bargańska et al., 2016; Niell et al., 2018) being essential the use of sentinel hives to monitor not only effects but also exposure to these compounds (EFSA Scientific Committee et al., 2021).

Based on the obtained data and considering the cost of residue analysis, the implemented xMAP acetamiprid immunoassay has shown to be a fast, medium-high throughput and reliable option to analyze a large set of diverse samples. Nonetheless, considering sampling efficiency (cost, time spent on sampling, and output), we defend that the analysis of colony products like honey and beebread through time can be used as a tool to evaluate landscape suitability regarding pesticides presence, while fresh pollen can provide a snapshot in time. Additionally, the LFD prototype has proven to be a reliable and useful tool for in-field and lab-based prescreening (Posthuma-Trumpie et al., 2009) for the presence of neonicotinoids in landscape and colony matrices. Beekeepers and other stakeholders can use it for a fast and cheap screening to assess the spread of residues in the landscape and evaluate if honeybees are bringing pesticides residues into the colonies. For now, this device is validated for detecting six neonicotinoids (imidacloprid, acetamiprid, clothianidin, thiacloprid, nitenpyram and imidaclothiz) in several bee-related matrices.

## 5 Conclusions

In this study, real landscape-based scenarios where acetamiprid is usually applied and beekeepers usually install their colonies were tested. The low ETR values found show that spraying events covering up to 22 % of the honey bees foraging ranges towards the end of the flowering period of the main flower resources present a negligible risk to the honeybee’ colonies. Nevertheless, these results must be interpreted with caution, since (1) in all study windows, honey bees had non-sprayed areas to forage; (2) no other crop fields with pesticides' input were inside the study areas, limiting cross contamination and possible synergistic effects caused by other chemicals (e.g., Wang et al., 2020); and (3) the study was performed in a period where nectar and pollen income might not be so high as during the peak of flowering.

The range of acetamiprid exposure was successfully accessed showing how acetamiprid residues behave in the landscape and follow their fate in honeybee colonies by measuring its accumulation on several colony matrices. On the other hand, no clear pattern was detected between the most exposed (near roads) flower resources and the ones inside the eucalyptus area, despite the low *p*-value (0.07).

The following are the supplementary data related to this article.

Supplementary material A Study windows location and sprayed area. Supplementary material A

Supplementary material B Honey bee colonies status before and after pesticide exposure. Supplementary material B

Supplementary material C Protocol used for the acetamiprid residues analysis. Supplementary material C

## CRediT authorship contribution statement

N.C., M.X., J.P., and J.P.S. conceived the study; N.C. and S.S. conducted the field experiments; M.X. and J.P. conducted the laboratory analysis; N.C. and J.P.S. analyzed the data; N.C., M.X., J.P., H.A.P. and J.P.S. wrote the manuscript. All authors read and reviewed the manuscript.

## Declaration of competing interest

The authors declare that they have no known competing financial interests or personal relationships that could have appeared to influence the work reported in this paper.
