# Supplementary material for: Exposure and risk assessment of acetamiprid in honey bee colonies under a real exposure scenario in Eucalyptus sp. landscapes
Source: Sci Total Environ. 2022 Sep 20;840:156485. doi: 10.1016/j.scitotenv.2022.156485 (PMC9247745; doi:10.1016/j.scitotenv.2022.156485)
Supplement: Supplementary material A — Study windows location and sprayed area. [file mmc1.docx]

Exposure and risk assessment of acetamiprid in honey bee colonies under a real exposure scenario in Eucalyptus sp. landscapes

*Supplementary material A*

Nuno Capela^a^, Mang Xu^b^, Sandra Simões^a^, Henrique Azevedo-Pereira^c^, Jeroen Peters^b^, José Paulo Sousa^a^

^a^ Centre for Functional Ecology, Department of Life Sciences, Associated Laboratory TERRA, University of Coimbra, Portugal;

^b^ Wageningen Food safety Research, Wageningen, The Netherlands;

^c^ ForestWISE - Collaborative Laboratory for Integrated Forest & Fire Management, Quinta de Prados, 5001-801 Vila Real, Portugal.

**Corresponding author**

nunocapela.bio@gmail.com

Departamento Ciências da Vida, Calçada Martin de Freitas, 3000-456 Coimbra, Portugal


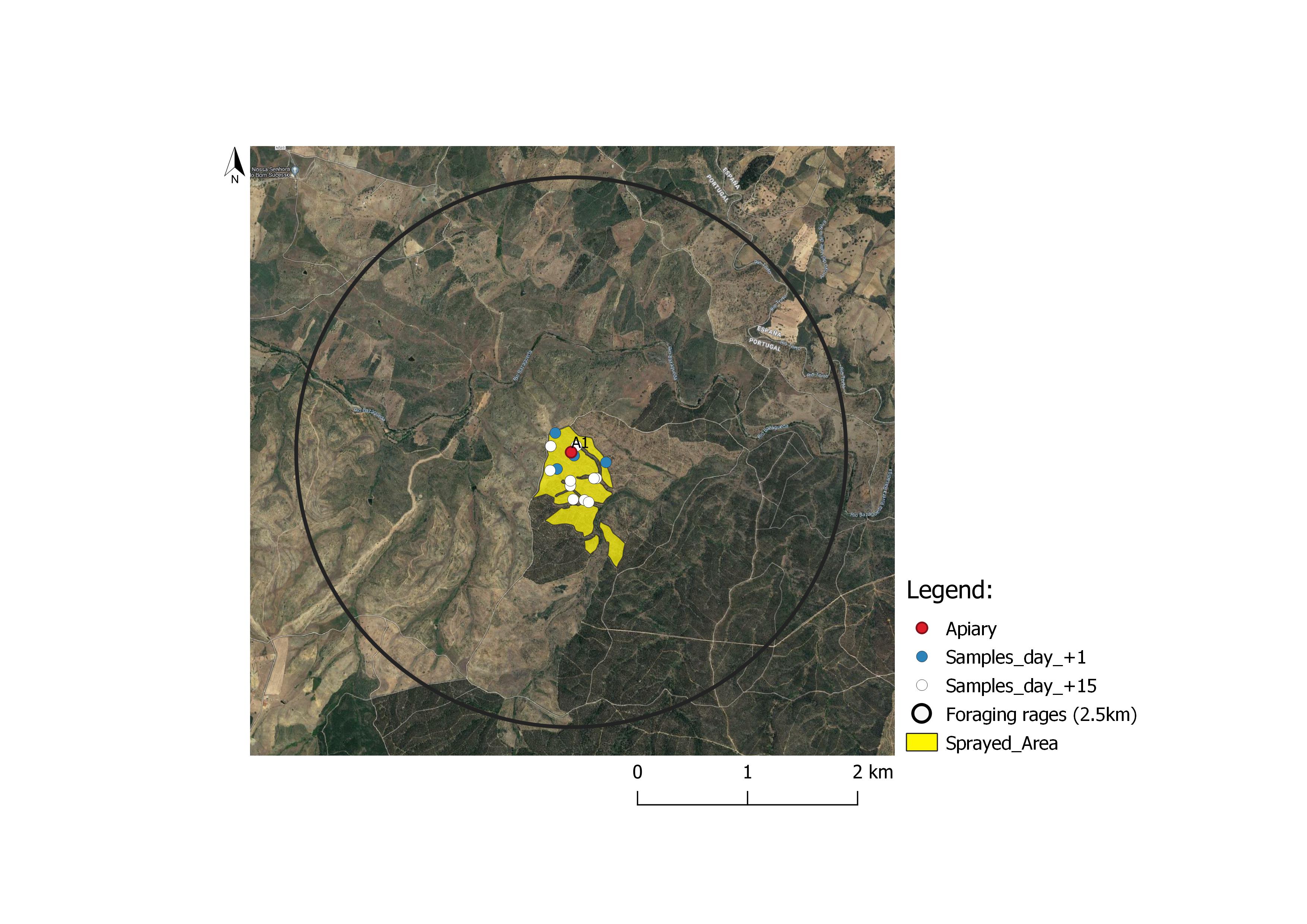
Figure SA1: Sprayed area (0.456 km^2^) and Study Window 1 (apiary A1) location.


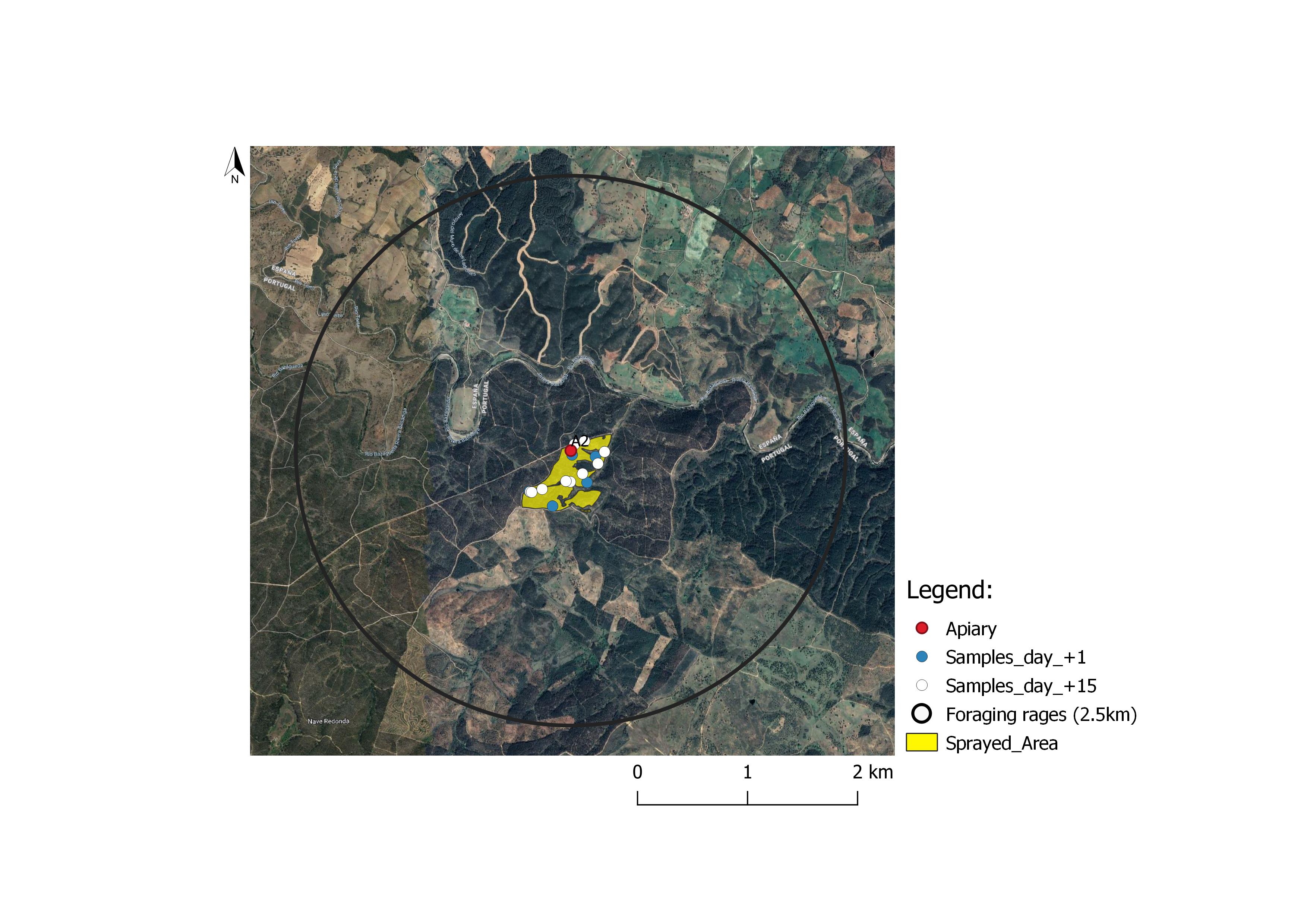
Figure SA2: Sprayed area (0.247 km^2^) and Study Window 2 (apiary A2) location.


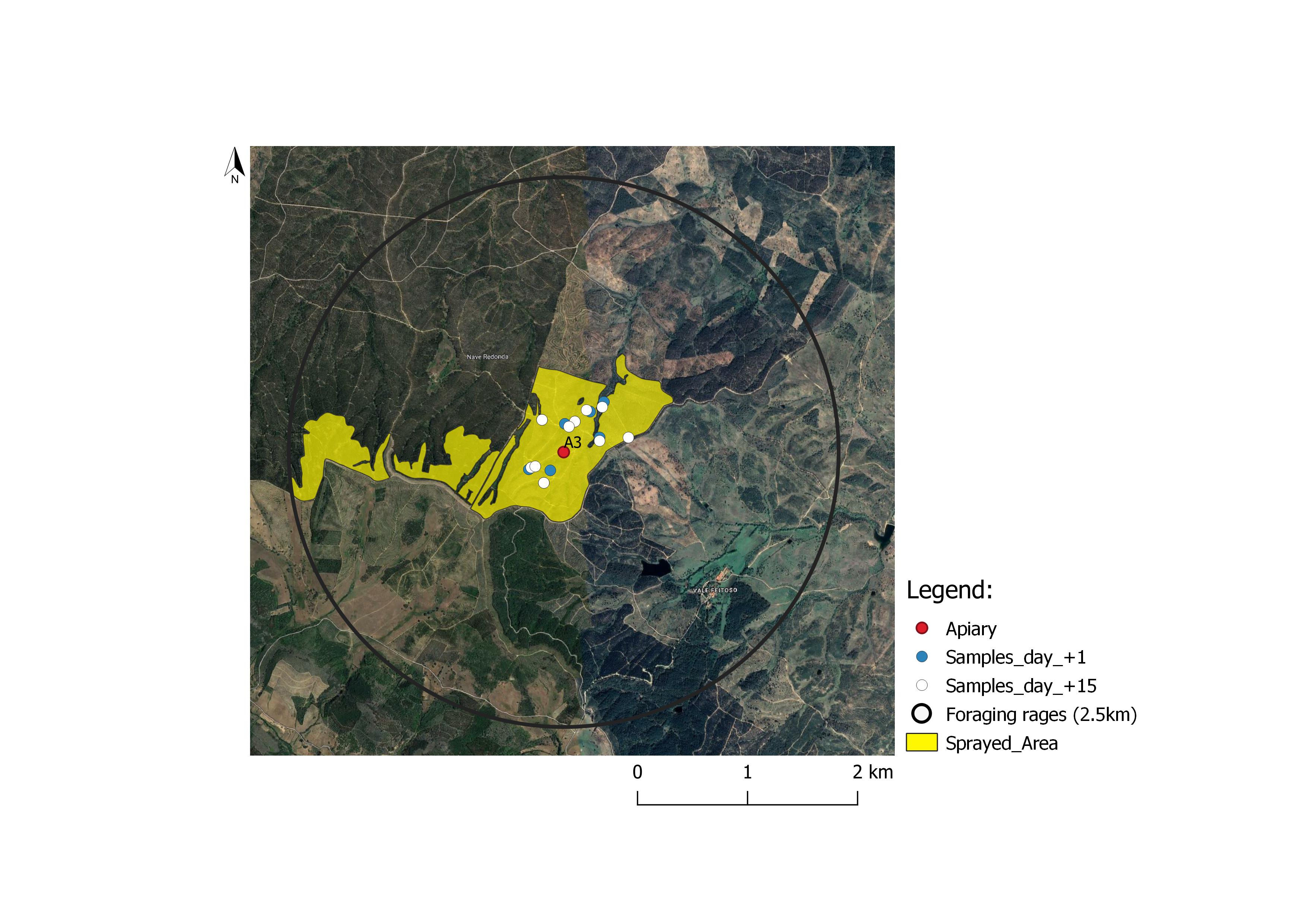


Figure SA3: Sprayed area (1.860 km^2^) and Study Window 3 (apiary A3) location.


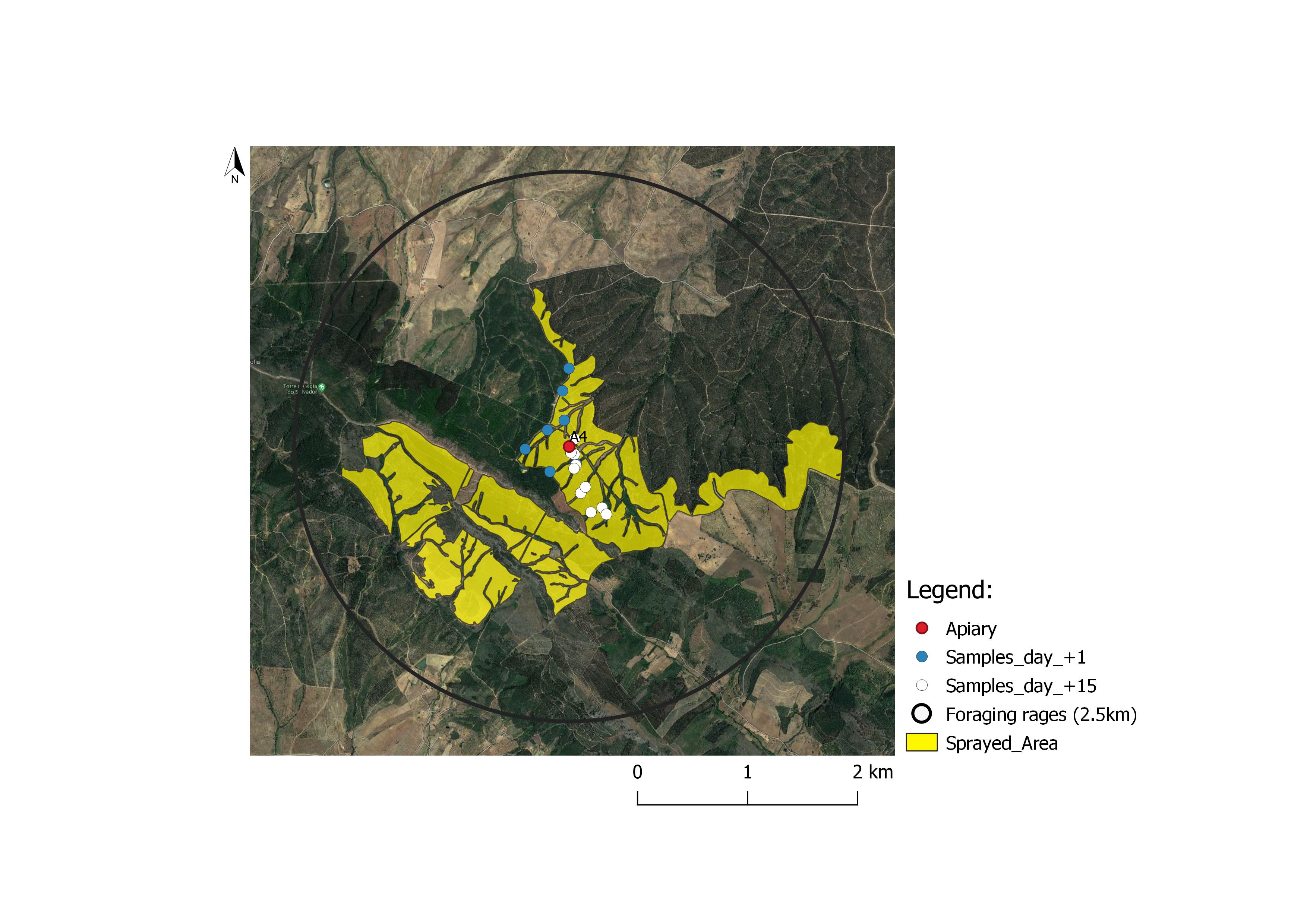
Figure SA4: Sprayed area (3.117 km^2^) and Study Window 4 (apiary A4) location.


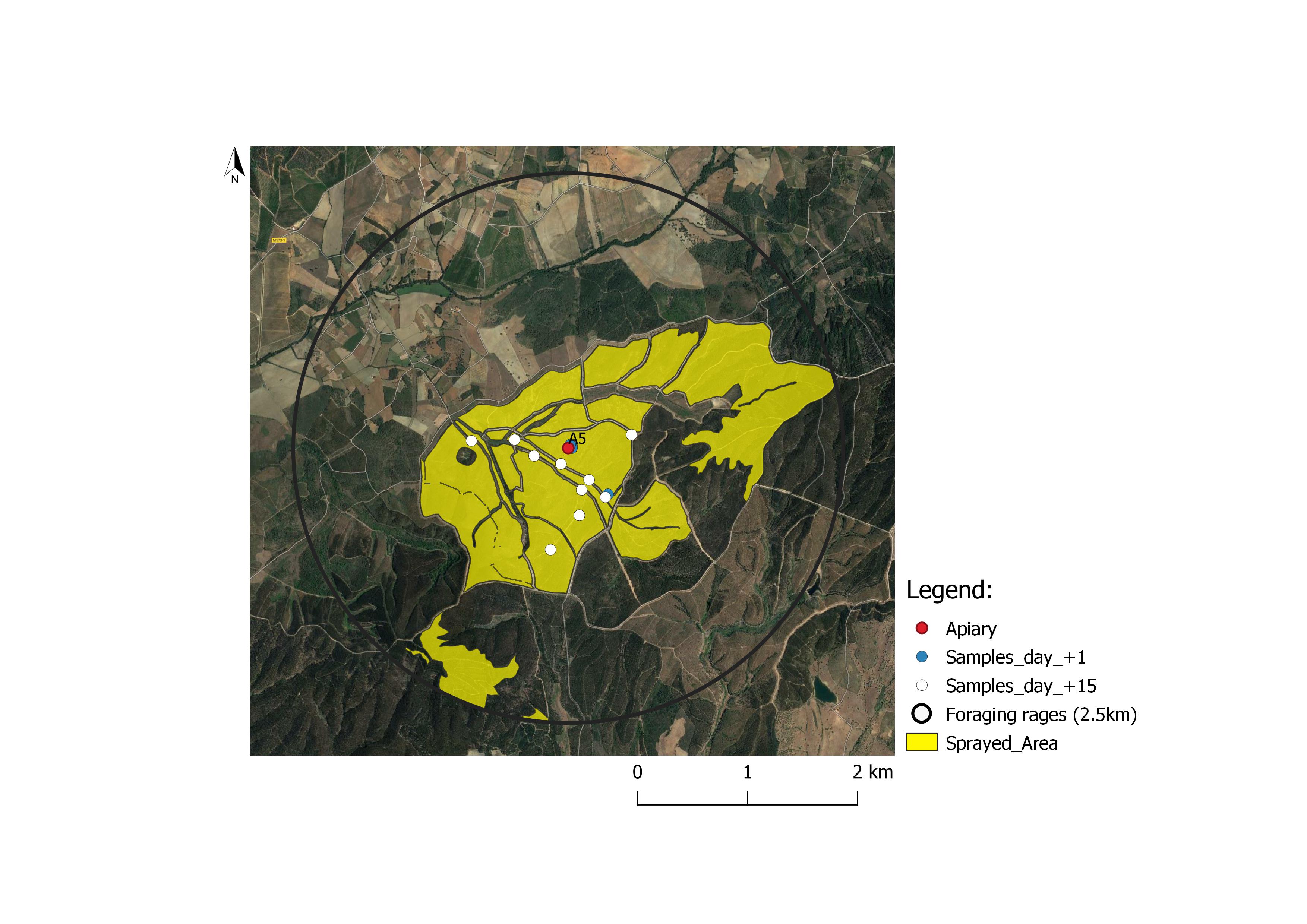
Figure SA5: Sprayed area (4.486 km^2^) and Study Window 5 (apiary A5) location.
