# Supplementary material for: Exposure and risk assessment of acetamiprid in honey bee colonies under a real exposure scenario in Eucalyptus sp. landscapes
Source: Sci Total Environ. 2022 Sep 20;840:156485. doi: 10.1016/j.scitotenv.2022.156485 (PMC9247745; doi:10.1016/j.scitotenv.2022.156485)
Supplement: Supplementary material B — Honey bee colonies status before and after pesticide exposure. [file mmc2.docx]

Exposure and risk assessment of acetamiprid in honey bee colonies under a real exposure scenario in Eucalyptus sp. landscapes

*Supplementary material B*

Nuno Capela^a^, Mang Xu^b^, Sandra Simões^a^, Henrique Azevedo-Pereira^c^, Jeroen Peters^b^, José Paulo Sousa^a^

^a^ Centre for Functional Ecology, Department of Life Sciences, Associated Laboratory TERRA, University of Coimbra, Portugal;

^b^ Wageningen Food safety Research, Wageningen, The Netherlands;

^c^ ForestWISE - Collaborative Laboratory for Integrated Forest & Fire Management, Quinta de Prados, 5001-801 Vila Real, Portugal.

**Corresponding author**

nunocapela.bio@gmail.com

Departamento Ciências da Vida, Calçada Martin de Freitas, 3000-456 Coimbra, Portugal

Table B1: Colony assessment was performed using the methods described in Dupont et al., 2021. Colonies were assessed one week before exposure (05-05-20) and 3 weeks after (07-06-20). For each colony, besides the visual assessment of disease symptoms, it was measured the number of adult bees (population), the amount (kg) of nectar/honey in the colony, and the number of brood (egg, larva and pupa) and beebread cells.

| Date | Apiary | Colony | Population | Honey and nectar (kg) | Number of brood cells | Number of beebread cells |
| --- | --- | --- | --- | --- | --- | --- |
| 05-05-20 | A1 | 3 | 16048 | 2.9 | 21570 | 4778 |
| 05-05-20 | A1 | 10 | 25403 | 3.3 | 21115 | 7201 |
| 05-05-20 | A2 | 5 | 16613 | 2.2 | 15601 | 3671 |
| 05-05-20 | A2 | 6 | 24919 | 3.9 | 21686 | 4992 |
| 05-05-20 | A3 | 1 | 24516 | 1.9 | 21926 | 3418 |
| 05-05-20 | A3 | 7 | 14919 | 2.4 | 21532 | 4325 |
| 05-05-20 | A4 | 4 | 17016 | 4.7 | 15852 | 4275 |
| 05-05-20 | A4 | 8 | 24839 | 2.4 | 18039 | 6173 |
| 05-05-20 | A5 | 2 | 16694 | 2.8 | 14003 | 6322 |
| 05-05-20 | A5 | 9 | 24597 | 1.6 | 24314 | 4982 |
| 07-06-20 | A1 | 3 | 21613 | 3.4 | 18109 | 7496 |
| 07-06-20 | A1 | 10 | 24355 | 5.7 | 13920 | 10491 |
| 07-06-20 | A2 | 5 | 15565 | 2.8 | 12683 | 4910 |
| 07-06-20 | A2 | 6 | 25242 | 8.9 | 17243 | 4704 |
| 07-06-20 | A3 | 1 | 18065 | 4.3 | 18300 | 7569 |
| 07-06-20 | A3 | 7 | 20968 | 7.1 | 20656 | 6786 |
| 07-06-20 | A4 | 4 | 20323 | 7.6 | 14825 | 4409 |
| 07-06-20 | A4 | 8 | 25887 | 8.2 | 15773 | 5407 |
| 07-06-20 | A5 | 2 | 16048 | 3.2 | 13074 | 6741 |
| 07-06-20 | A5 | 9 | 22984 | 6.6 | 22107 | 5753 |
