# Supplementary material for: Exposure and risk assessment of acetamiprid in honey bee colonies under a real exposure scenario in Eucalyptus sp. landscapes
Source: Sci Total Environ. 2022 Sep 20;840:156485. doi: 10.1016/j.scitotenv.2022.156485 (PMC9247745; doi:10.1016/j.scitotenv.2022.156485)
Supplement: Supplementary material C — Protocol used for the acetamiprid residues analysis. [file mmc3.docx]

Exposure and risk assessment of acetamiprid in honey bee colonies under a real exposure scenario in Eucalyptus sp. landscapes

*Supplementary material C*

Nuno Capela^a^, Mang Xu^b^, Sandra Simões^a^, Henrique Azevedo-Pereira^c^, Jeroen Peters^b^, José Paulo Sousa^a^

^a^ Centre for Functional Ecology, Department of Life Sciences, Associated Laboratory TERRA, University of Coimbra, Portugal;

^b^ Wageningen Food safety Research, Wageningen, The Netherlands;

^c^ ForestWISE - Collaborative Laboratory for Integrated Forest & Fire Management, Quinta de Prados, 5001-801 Vila Real, Portugal.

**Corresponding author**

nunocapela.bio@gmail.com

Departamento Ciências da Vida, Calçada Martin de Freitas, 3000-456 Coimbra, Portugal

**xMAP technology for flowers and bee products residue analysis**

A bead-based array platform (xMAP multi-analyte-profiling) (Hsu et al., 2009) has proven to be a viable option to semi-quantify a large amount of field samples. xMAP technology has been broadly applied in various fields, including food safety, environmental pollutant monitoring and medical diagnostics (Hamza et al., 2014; Peters et al., 2014; Pan et al., 2019). One of the first successful attempts of utilizing xMAP in pesticide detection was a competitive, single-plex assay for detecting triazophos in vegetable matrices, which was further incorporated into a multiplex assay, screening triazophos, chlorpyrifos and carbofuran simultaneously in vegetable matrices (Guo et al., 2013; Liang et a., 2013). Nonetheless, these analyses require some technical and laborious procedures: samples must be collected, properly stored, transported to, and analyzed in the laboratory. The use of equipment – in situ – that could reveal the presence (screening) of specific pesticides in environmental samples can give an important notice of what PPPs might be applied in a specific location. The use of lateral flow devices (LFDs) can be the answer to this quest. In LFDs, an immunoassay is powered by capillary force and uses a similar principal as a competitive enzyme-linked immunosorbent assay (ELISA): specific antibodies are used for biorecognition, and the LFD shows a colorimetric display as its readout (Posthuma-Trumpie et al., 2019). Over the years, paper-based LFDs were developed for a wide range of applications for rapid screening at the point of need (Anfossi et al., 2013; Huang et al., 2016). In 2017, Wang et al. developed a quantum-dot-based LFD for the fast screening of three neonicotinoids (imidacloprid, clothianidin and imidaclothiz) in tea leaves. In 2019, the same group published an LFD for screening acetamiprid in agricultural products using the same method (Liu et al., 2019). These LFDs were claimed to be portable and sensitive (limit of detection at 1 ng/ml), with a simple extraction method which only uses boiling water. This makes fully on-site sample preparation and measurement realistic (Wang et al., 2017; Liu et al., 2019).

To analyze the samples generated from the field experiment, we chose a paper-based rapid LFD screening immunoassay with on-site applicability and a lab-based, matrix matched semi-quantitative xMAP immunoassay, both for the detection of acetamiprid. These immunoassays were effective with only a simple hot water extraction. The lateral flow screening assay has proven to be a fast, cheap, sensitive, qualitative and reliable pre-screening method. The dual channel LFD prototype produced by Zhejiang University with a previously determined limit of detection (LOD) of 10 ng/ml has confirmed these capabilities for screening purposes in this study, and are able to detect acetamiprid below 5 ppb in the most matrix extracts (bees, pollen, bee bread and eucalyptus leaves) and below 15 ppb in the bush flowers. The Ace-mAb used for this study showed high sensitivity to acetamiprid not only in the LFD analysis, it also provided an LOD of 0.08 ng/ml in the bead-based xMAP technology (unpublished data). Despite the fact that the LFD allows easy, robust but sensitive detection of acetamiprid, its readout is only visual in this experiment and therefore is not capable of quantification. Since our field experiment produced in total 366 different environmental samples, the LFD was only implemented on a subset of samples. For the semi-high throughput acetamiprid detection in the experimental samples, xMAP technology enabled rapid semi-quantification of a large number of samples with relatively low cost and manual work when compared to instrumental analysis (Postuma-Trumpie et al., 2009). Although the planar array analyzer is not intentionally designed for on-site analysis, it is still portable and can be easily set up in a minimal or mobile lab environment. A wide range of samples were suitable for direct detection using the matrix-matched calibration curves. However, some samples were highly contaminated and needed dilution steps to be able to quantify. Throughout the qualitative and semi-quantitative analyses of these field samples, the matrix effect rises as the most challenging: In bees, pollen and beebread, the main matrix components are protein and fat, while in nectar, sugar is the main matrix factor. In plants, odorous essential oils as well as color pigments can be extracted together with the pesticides and introduce matrix effect.

**Instrumentation and Materials**

A MAGPIX planar array analyzer, drive fluid and MagPlex #038 paramagnetic microspheres were purchased from Luminex (Austin, USA). Acetamiprid-monoclonal antibody (Ace-mAb) used for detection and the acetamiprid-ovalbumin (Ace-OVA) conjugate used for paramagnetic microsphere coupling were generously provided by Institute of Pesticide and Environmental Toxicology Zhejiang University. The Goat-anti-mouse IgG-R-Phycoerythrin conjugate (GAM-RPE) was obtained from Moss (Pasadena, USA). The xMAP assays were performed in Cellstar flat-bottomed 96-well microtiter plates purchased from Greiner Bio-One B.V. (Alphen aan den Rijn, the Netherlands). An electric household kettle (TOMADO) was used, for the hot water acetamiprid extraction of all samples. An Ystral polytron (Ballrechten-Dottingen, Germany) was used for homogenizing bee samples, while Whatmann 5951/2 filter paper (GE Healthcare, Germany) and 1.2 µm Durapore membrane filter plates (Millipore, Darmstadt, Germany) were used for filtering the extracts. PBS, in dissolvable pellets, was also purchased from Millipore, while MES hydrate, N-Hydroxysulfosuccinimide sodium, 1-ethyl-3-(3-dimethylaminopropyl) carbodiimide hydrochloride, bovine serum albumin (BSA) and Tween-20 and the acetamiprid standard (purity: 99.9%) were all purchased from Sigma (Steinheim, Germany). The imidacloprid/acetamiprid dual channel Lateral Flow Devices (LFDs) were kindly produced on request by the Institute of Pesticide and Environmental Toxicology of Zhejiang University. Graphpad Prism version 8.0.0 for Windows (GraphPad Software, San Diego, USA) was used for data analysis.

**Microsphere coupling**

The #038 Magplex bead stock (1.25x10^7^ microspheres/ml) was thoroughly suspended by 10-minute vortexing. Next, 6.25x10^6^ microspheres were washed with distilled water. For activation, the microspheres were resuspended in 80 µl 100 mM monobasic sodium phosphate (pH 6.2), followed by the addition of 10 µl (500 µg) freshly dissolved N-Hydroxysulfosuccinimide sodium in deionized water and 10 µl (500 µg) freshly dissolved 1-ethyl-3-(3-dimethylaminopropyl) carbodiimide hydrochloride (in deionized water). This mixture was incubated for 20 minutes with gentle mixing every 5 minutes. 100 µg/ml Ace-mAb was prepared by diluting a 1 mg/ml stock (in PBS) with 50 mM MES buffer pH 5.0 and was allowed to react with the activated microspheres for two hours while gently mixing. The excess antibodies were removed, and the microspheres were blocked with PBS-TBN buffer (1x PBS, 0.1% BSA, 0.02% Tween-20, 0.05% NaN_3_) for 30 minutes while gently mixing. The Ace-OVA coupled microspheres were stored in 200 µl PBS-TBN at 4^o^C in the dark until further use.

**Sample preparation**

For pollen, nectar, bee bread, plant leaves and flowers, 1 g of material was extracted with 10 ml boiling tap water for 30 minutes. Extracts were vigorously shaken every 5 minutes to ensure optimal extraction. For bee samples, 1 g of bees was weighed in a 50 ml tube, and 10 ml boiling tap water was added. Additionally, the bees were homogenized with a polytron. All crude extracts were filtered through filter paper and the flow through was collected. A subset of the extracts was used for LFD analysis, while all extracts were used for a xMAP planar array analysis. To control the samples for possible pre-existing acetamiprid contamination, Day -1 samples were measured on-site at the apiary via LFDs.

**LFD analysis**

For this project, prototype dual channel LFDs for the detection of imidacloprid and acetamiprid were applied on-site in the field and in the lab for the detection of acetamiprid. These prototype LFDs contain the same Ace-mAb and Ace-OVA as used in the xMAP assay. Like the xMAP method, the LFD uses an inhibition assay principle (Fig. S1a)

Extracts were prepared as described previously. After the extract cooled down to room temperature, two drops of the sample extracts were added to each sample well of the LFD using a Pasteur pipette and allowed to develop for 15 minutes before visual read-out. After read-out, positive or negative results were determined (Fig. S1b and S1c).


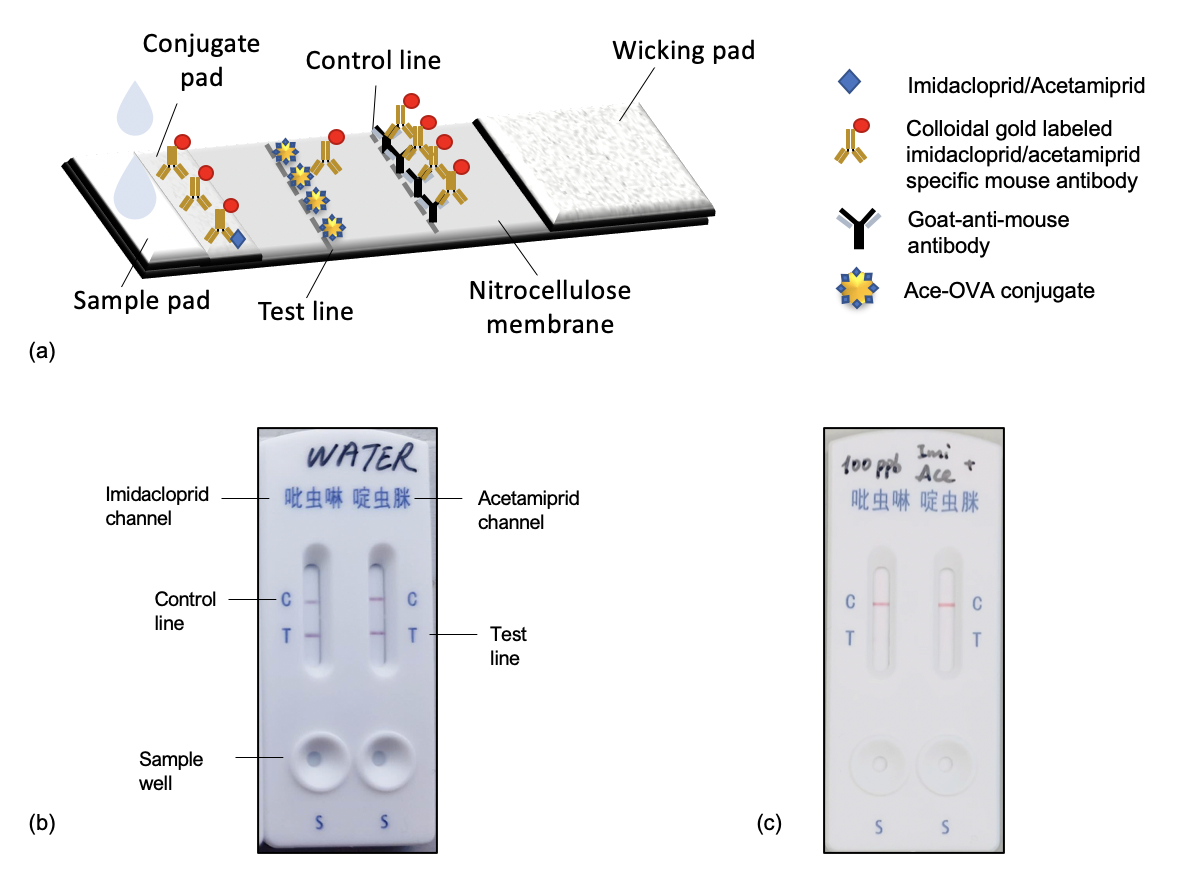


Fig. SC1: (a) Working principle of the LFD. Control line (goat-anti-mouse antibody) and test line (Imi-OVA or Ace-OVA conjugate) are immobilized on a nitrocellulose membrane. Colloidal gold coupled imidacloprid/acetamiprid specific antibody, dried on the conjugate pad flows throughout nitrocellulose membrane via capillary flow. In a valid assay, the specific reporter antibody on the gold nanoparticles are recognized by the anti-mouse antibodies on the control line, thus the control lines should always be present as a part of a valid readout. When testing a positive sample, the analytes in the sample compete for imidacloprid/acetamiprid specific antibodies against the Imi-OVA/Ace-OVA on the test line, and therefore causing faint or absence test lines. Fig. adapted from Liu et al. (2019) and Wang et al. (2017). (b) The imidacloprid/acetamiprid dual channel LFD applied with tap water sample. Since there is no neonicotinoid present in the water, control and test lines are clearly visible. (c) Readout of the dual channel LFD after application of water samples containing 100 ng/ml imidacloprid and 100 ng/ml acetamiprid. The free imidacloprid and acetamiprid occupy the specific antibodies and prevent their binding to the control line, resulting the absence of the test lines.

**Development of the acetamiprid screening assay**

To determine the optimal working concentration of the Ace-mAb, 2-fold serial dilutions of the Ace-mAb were prepared in 1x PBST buffer (0.01 M, 0.1% BSA and 0.02% Tween-20, pH 7.4) in a 96 well microtiter plate in duplicate. The serial dilution of Ace-mAb was incubated with 10 µl microsphere solution (approximately 1000 microspheres, in 1x PBST buffer) for 20 minutes while gently shaking. The excess Ace-mAb was washed off, and 100 µl GAM-RPE (2 µg/ml) was added as a secondary reporter antibody and incubated for another 20 minutes. The excess GAM-RPE was removed prior to the measurement. Read out was performed by the MAGPIX planar array analyzer operating on Luminex xPONENT software version 4.3. The minimum count was set to 50 microspheres. The mean fluorescent intensity (MFI) was measured and plotted against the Ace mAb concentration. The optimal concentration of Ace mAb in this assay format was determined by the corresponding concentration at 1000 MFI.

**Sample screening and determination of the sensitivity**

Dose-response curves of acetamiprid, in the targeted matrices, ranging from 100 ng/ml to 0.01 ng/ml, were prepared by addition of acetamiprid to the blank matrix. For this purpose, the tested Day-1 samples were selected. As a negative control, and zero value, the blank matrix extracts were used. The samples and the calibration curve were filtered through 1.2 µm filter plates to remove disturbing matrix particles. Next, the dose-response curves and samples were measured in duplicate. In short, Ace-OVA coupled microspheres were diluted 1:100 with 10x PBST buffer (0.1 M, 1% BSA and 0.2% Tween-20, pH 7.4), while Ace-mAb was diluted to 10x optimal dilution in 1x PBST buffer. 10 µl of diluted microsphere solution (approximately 1000 microspheres) and 10 µl of diluted Ace mAb were incubated with 90 µl of filtered extracts or standards. This mixture was incubated for 20 minutes, and the assay was developed and measured in duplicates as previously described.

**xMAP planar array data processing**

Each MFI reading was normalized against the MFI of the corresponding blank matrix (B/B0). The B/B0 values of the dose-response curves were plotted against the acetamiprid concentrations, on a logarithmic scale. Four-parameter logistics nonlinear regression was implemented for curve fitting the unknown samples on the respective acetamiprid dose-response curves using Graphpad Prism version 8.0.0 for Windows. The acetamiprid concentrations in the unknown samples were calculated by the same software. The sample extracts that had concentrations above the dose-response curve’s upper limit were diluted and remeasured together with the standards prepared in the correspondingly diluted matrices if necessary.

**References**

Peters, J., Cardall, A., Haasnoot, W., & Nielen, M. W. (2014). 6-Plex microsphere immunoassay with imaging planar array detection for mycotoxins in barley. Analyst, 139(16), 3968-3976.

Posthuma-Trumpie, G. A., Korf, J., & van Amerongen, A. (2009). Lateral flow (immuno) assay: its strengths, weaknesses, opportunities and threats. A literature survey. Analytical and bioanalytical chemistry, 393(2), 569-582.

Wang, S., Liu, Y., Jiao, S., Zhao, Y., Guo, Y., Wang, M., & Zhu, G. (2017). Quantum-dot-based lateral flow immunoassay for detection of neonicotinoid residues in tea leaves. Journal of agricultural and food chemistry, 65(46), 10107-10114.

Anfossi, L., Baggiani, C., Giovannoli, C., D’Arco, G., & Giraudi, G. (2013). Lateral-flow immunoassays for mycotoxins and phycotoxins: a review. Analytical and bioanalytical chemistry, 405(2), 467-480.

Huang, X., Aguilar, Z. P., Xu, H., Lai, W., & Xiong, Y. (2016). Membrane-based lateral flow immunochromatographic strip with nanoparticles as reporters for detection: A review. Biosensors and Bioelectronics, 75, 166-180.

Liu, Y., Zhao, Y., Zhang, T., Chang, Y., Wang, S., Zou, R., ... & Guo, Y. (2019). Quantum dots-based immunochromatographic strip for rapid and sensitive detection of acetamiprid in agricultural products. Frontiers in chemistry, 7, 76.

Hsu, H. Y., Joos, T. O., & Koga, H. (2009). Multiplex microsphere‐based flow cytometric platforms for protein analysis and their application in clinical proteomics–from assays to results. Electrophoresis, 30(23), 4008-4019.

Pan, J., Zheng, Q. Z., Li, Y., Yu, L. L., Wu, Q. W., Zheng, J. Y., ... & Huang, Y. (2019). Discovery and validation of a serologic autoantibody panel for early diagnosis of esophageal squamous cell carcinoma. Cancer Epidemiology and Prevention Biomarkers, 28(9), 1454-1460.

Hamza, I. A., Jurzik, L., & Wilhelm, M. (2014). Development of a Luminex assay for the simultaneous detection of human enteric viruses in sewage and river water. Journal of virological methods, 204, 65-72.

Liang, C., Zou, M., Guo, L., Gui, W., & Zhu, G. (2013). Development of a bead-based immunoassay for detection of triazophos and application validation. Food and agricultural immunology, 24(1), 9-20.

Guo, Y., Tian, J., Liang, C., Zhu, G., & Gui, W. (2013). Multiplex bead-array competitive immunoassay for simultaneous detection of three pesticides in vegetables. Microchimica Acta, 180(5), 387-395.
